# Paradoxical activation of MEK/ERK signaling induced by B-Raf inhibition enhances DR5 expression and DR5 activation-induced apoptosis in Ras-mutant cancer cells

**DOI:** 10.1038/srep26803

**Published:** 2016-05-25

**Authors:** You-Take Oh, Jiusheng Deng, Ping Yue, Shi-Yong Sun

**Affiliations:** 1 Department of Hematology and Medical Oncology, Emory University School of Medicine and Winship Cancer Institute, Atlanta, Georgia, USA

## Abstract

B-Raf inhibitors have been used for the treatment of some B-Raf–mutated cancers. They effectively inhibit B-Raf/MEK/ERK signaling in cancers harboring mutant B-Raf, but paradoxically activates MEK/ERK in Ras-mutated cancers. Death receptor 5 (DR5), a cell surface pro-apoptotic protein, triggers apoptosis upon ligation with tumor necrosis factor-related apoptosis-inducing ligand (TRAIL) or aggregation. This study focused on determining the effects of B-Raf inhibition on DR5 expression and DR5 activation-induced apoptosis in Ras-mutant cancer cells. Using chemical and genetic approaches, we have demonstrated that the B-Raf inhibitor PLX4032 induces DR5 upregulation exclusively in Ras-mutant cancer cells; this effect is dependent on Ras/c-Raf/MEK/ERK signaling activation. PLX4032 induces DR5 expression at transcriptional levels, largely due to enhancing CHOP/Elk1-mediated DR5 transcription. Pre-exposure of Ras-mutated cancer cells to PLX4032 sensitizes them to TRAIL-induced apoptosis; this is also a c-Raf/MEK/ERK-dependent event. Collectively, our findings highlight a previously undiscovered effect of B-Raf inhibition on the induction of DR5 expression and the enhancement of DR5 activation-induced apoptosis in Ras-mutant cancer cells and hence may suggest a novel therapeutic strategy against Ras-mutated cancer cells by driving their death due to DR5-dependent apoptosis through B-Raf inhibition.

B-Raf mutation, an oncogenic driver mutation, frequently occurs in certain types of cancers such as melanoma (50–80% of cases), papillary thyroid carcinoma (~45%), hepatocellular carcinoma (~40%) and colorectal cancer (~10%)^1^,^2^. The most frequent mutation occurs in the kinase domain with valine being replaced by glutamic acid at codon 600 (V600E), leading to constitutive activation of B-Raf kinase and downstream MEK/ERK signaling^2^. These findings have spurred the effort to develop B-Raf inhibitors as anticancer drugs. As a result, several selective B-Raf (V600E) inhibitors including PLX4032 (vemurafenib) and dabrafenib (Tafinlar) have been developed and tested in the clinic^2^,^3^,^4^. The recent success of B-Raf inhibitors in the treatment of advanced melanoma harboring mutant B-Raf has encouraged further research into the potential applications of B-Raf-targeted therapy in other cancer types^2^,^5^. Unfortunately, relapse or resistance occurs within months although these B-Raf inhibitors have shown evident efficacy in patients with melanoma with the B-Raf V600E mutation^6^,^7^. Several underlying mechanisms have been proposed; however the major cause of resistance is associated with the paradoxical activation of MEK/ERK signaling^6^,^7^,^8^, primarily due to c-Raf activation^9^,^10^,^11^. This paradoxical activation of MEK/ERK signaling makes B-Raf–mutant cancers insensitive to treatment with B-Raf inhibitors, resulting in diminished therapeutic efficacy. Accordingly, the combination of B-Raf and MEK inhibitors has been emerged as a strategy to delay or prevent the development of resistance and increase of secondary cancers, by preventing this paradoxical activation of MEK/ERK signaling^1^,^7^,^8^.

Death receptor 5 (DR5; also called TRAIL-R2 or killer/DR5) is a cell surface receptor for the death ligand tumor necrosis factor-related apoptosis-inducing ligand (TRAIL). Human cells have another homologue of TRAIL receptor known as death receptor 4 (DR4; also called TRAIL-R1), whereas in mouse cells only one TRAIL receptor is present. Several types of immune cells including natural killer (NK) cells, T cells, natural killer T (NKT) cells, dendritic cells and macrophages expresses endogenous TRAIL^12^. Ligation of TRAIL (or recombinant TRAIL) to its functional death receptors (e.g., DR5) leads to recruitment of the adaptor protein Fas-associated death domain (FADD) to the cytoplasmic region of receptor followed by recruitment of procaspase-8 or procaspase-10. This complex formation triggers cleavage and activation of caspase-8 or caspase-10, which in turn activates downstream caspase-3, -6, -7 and causes eventual apoptosis^13^,^14^. Induction of apoptosis caused by endogenous TRAIL binding to its receptors (e.g., TRAIL/DR5) is thus a critical underlying mechanism of the immune surveillance of tumors and metastases^15^,^12^. Moreover, induction of DR5 aggregation or trimerization, e.g., by a DR5 agonistic antibody, also induces apoptosis. This provides scientific rationale for developing pharmacological DR5 agonistic antibodies, some of which have been tested in the clinic as potential cancer therapeutics^16^. Thus, soluble recombinant TRAIL and DR5 agonistic antibodies represent potential anticancer therapeutics^14^,^17^,^18^.

Our previous studies have demonstrated that Ras/Raf/MEK/ERK signaling increases CHOP- and Elk-dependent DR5 expression^19^,^20^. Inhibition of B-Raf or MEK in B-Raf–mutant cancer cells suppresses ERK activation accompanied by downregulation of DR5 expression and decreased cell sensitivity to DR5 activation-induced apoptosis, as we recently demonstrated^21^. This finding further supports the predominant role of MEK/ERK signaling in the positive regulation of DR5 expression. The current study focuses on determining the impact of B-Raf inhibition on DR5 expression and DR5 activation-induced apoptosis in Ras-mutant cancer cells. We hypothesized that B-Raf inhibition in Ras-mutant cancer cells will increase DR5 expression and enhance cell response to DR5 activation-induced apoptosis due to the paradoxical activation of MEK/ERK signaling.

## Results

### Pharmacological inhibition of B-Raf activates ERK and increases DR5 expression exclusively in Ras-mutant cancer cell lines

**Figure 1:**
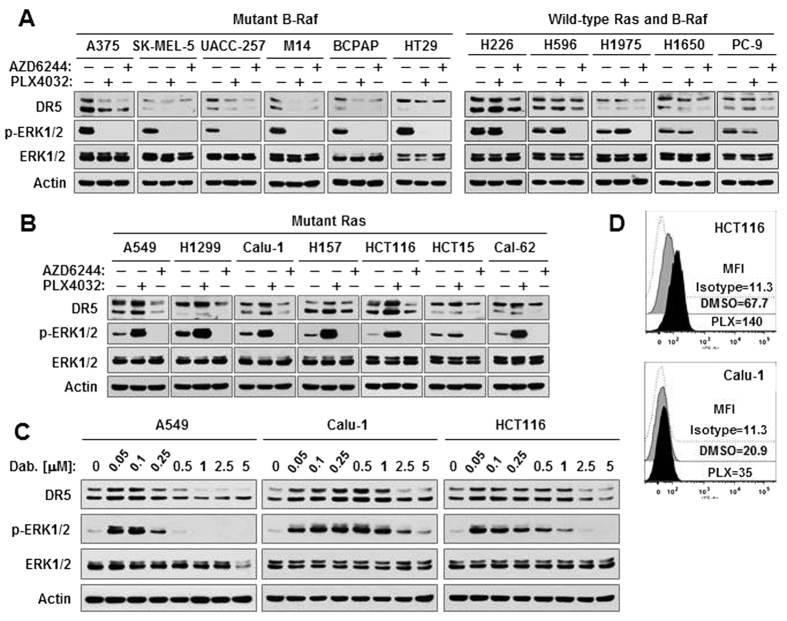
Pharmacological inhibition of B-Raf induces paradoxical activation of ERK and DR5 expression in Ras-mutant cancer cell lines (**B–D**), but not in B-Raf–mutant cell lines or cell lines without these mutations (**A**). (**A**,**B**) The indicated cancer cell lines were treated with 5 μM PLX4032 or AZD6244 for 12 h and then collected for preparation of whole-cell protein lysates and subsequent Western blotting. (**C)**, Different cancer cell lines we indicated were exposed to various concentrations of dabrafenib (Dab.) for 12 h and then collected for preparation of whole-cell protein lysates and subsequent Western blotting. (**D)**, The indicated cell lines were exposed to 10 μM PL4032 (PLX) for 24 h and then harvested for detection of cell surface DR5 uisng flow cytometry. Mean fluorescent intensity (MFI) for each sample was indicated.

We first compared the effects of PLX4032 with AZD6244 (a MEK inhibitor) on ERK activation and DR5 expression in various cancer cell lines with B-Raf mutation, Ras (K-Ras and N-Ras) mutation or wild-type (WT) B-Raf and Ras. As previously reported^21^, both inhibitors decreased the levels of p-ERK1/2 and DR5 in cancer cell lines carrying mutant B-Raf gene (Fig. 1A). In contrast, PLX4032 increased the levels of both p-ERK1/2 and DR5 in the tested cancer cell lines harboring either mutant K-Ras or N-Ras (H1299), whereas AZD6244 was still effective in suppressing ERK phosphorylation and DR5 expression (Fig. 1B). In cancer cell lines without these mutations, PLX4032 had minimal or no effects on ERK phosphorylation and DR5 expression while AZD6244 continued to decrease ERK phosphorylation and DR5 expression (Fig. 1A). We also determined whether dabrafenib, another clinically used B-Raf (V600E) inhibitor, exerts similar effects in Ras-mutant cancer cell lines. At low concentration ranges between 50 nM and 1000 nM, dabrafenib increased p-ERK1/2 levels accompanied with elevation of DR5 in three representative Ras-mutant cell lines (Fig. 1C). These results demonstrate that B-Raf inhibition (e.g., with PLX4032 or dabrafenib) activates ERK and increases DR5 expression exclusively in Ras-mutant cancer cell lines. Beyond the increase in total levels of DR5 protein, PLX4032 also elevated cell surface DR5 levels in the representative Ras-mutant cancer cell lines (Fig. 1D).

### B-Raf inhibition-induced DR5 expression in Ras-mutant cancer cells is dependent on c-Raf/MEK/ERK signaling activation

**Figure 2:**
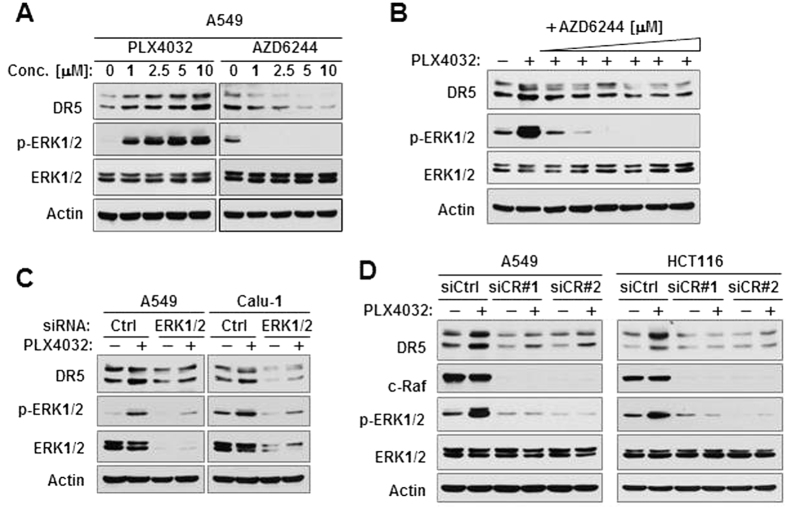
B-Raf inhibition-induced DR5 expression in Ras-mutant cancer cells is dependent on c-Raf/MEK/ERK signaling activation. (**A)**, A549 cells were exposed to the indicated concentrations of the inhibitors for 12 h. (**B)**, A549 cells were treated with 5 μM PLX4032 alone or in combination with increasing concentrations (0.01, 0.1, 0.5, 1, 2.5, and 5 μM) of AZD6244 for 12 h. (**C,D)**, The given cancer cell lines were transfected with different siRNAs. After 30 h, the cells were exposed to DMSO or 5 μM PLX4032 for another 12 h. After the aforementioned treatments, whole-cell protein lysates were prepared from the cells for detection of the indicated proteins with Western blotting.

To determine whether PLX4032-induced DR5 expression is secondary to MEK/ERK activation, we further compared the effects of PLX4032 and AZD6244 in a detailed way using multiple concentrations in Ras-mutant A549 cells and confirmed their opposing effects on ERK phosphorylation and DR5 expression; i.e., PLX4032 increased ERK phosphorylation and DR5 expression, whereas AZD6244 suppressed ERK phosphorylation and DR5 expression (Fig. 2A). In the presence of AZD6244, PLX4032 failed to increase p-ERK1/2 levels and DR5 expression (Fig. 2B). Consistently, inhibition of ERK by knocking down ERK1/2 expression blocked PLX4032-induced DR5 expression (Fig. 2C). These results indicate that PLX4032 induces MEK/ERK-dependent DR5 upregulation. It has been suggested that B-Raf inhibition in Ras-mutant cells causes c-Raf-dependent MEK/ERK activation^7^,^22^. Therefore we further determined whether c-Raf is involved in PLX4032-induced ERK activation and DR5 upregulation in Ras-mutant cancer cells. Using two different c-Raf siRNAs, we found that inhibiting c-Raf by knocking down its expression effectively abrogated the ability of PLX4032 to increase ERK phosphorylation and DR5 expression (Fig. 2D). Hence it is clear that PLX4032 induces c-Raf-dependent MEK/ERK activation and eventual DR5 upregulation in Ras-mutant cancer cells.

### B-Raf inhibition-induced DR5 expression in Ras-mutant cancer cells is Ras-dependent

**Figure 3:**
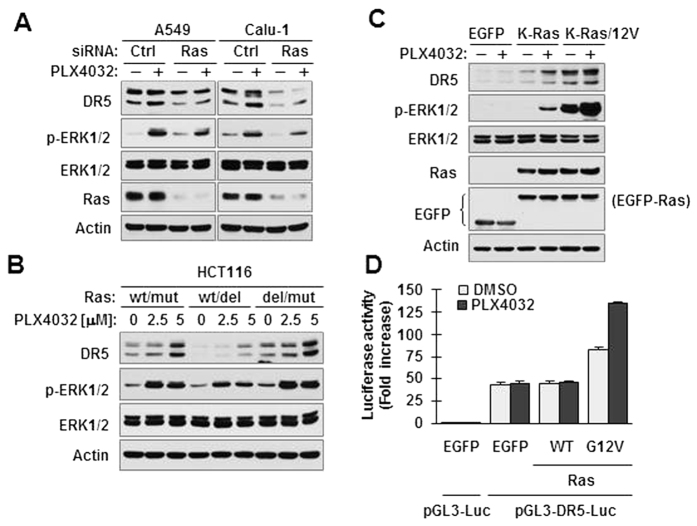
Knockdown or deletion of mutant K-Ras gene inhibits the ability of PLX4032 to activate ERK and increase DR5 expression (**A,B**), whereas enforced expression of mutant K-Ras enhances ERK activation and DR5 induction by PLX4032 (**C**,**D**). (**A)**, A549 or Calu-1 cells were transfected with the indicated siRNAs. After 30 h, the cells were exposed to 5 μΜ PLX4032 for 12 h. (**B)**, The indicated K-Ras knockout isogenic HCT116 cell lines were exposed to the given concentrations of PLX4032 for 12 h. (**C)**, HEK293T cells were transfected with the expression plasmids carrying the given K-Ras genes for 24 h and then exposed to 5 μM PLX4032 for an additional 10 h. After the aforementioned treatments (**A–C**), whole-cell protein lysates were prepared from the cells for detection of the indicated proteins with Western blotting. (**D)**, HEK293T cells were co-transfected with DR5 reporter construct and K-Ras expression plasmid for 26 h and then exposed to 5 μM PLX4032 for an additional 6 h before the cells were harvested for luciferase assay. The data represent means ± SDs of triplicate determinations.

To determine whether the presence of mutant Ras is required for PLX4032 to activate ERK and increase DR5 expression, we genetically knocked down Ras expression in two Ras-mutant cell lines and then examined its impact on PLX4032-induced ERK activation and DR5 expression. As presented in Fig. 3A, PLX4032 increased ERK phosphorylation and DR5 expression in control siRNA-transfected cells, but not or minimally in Ras siRNA-transfected cells, suggesting a Ras-dependent mechanism. In HCT116 cells possessing one allele of WT K-Ras and one allele of mutant K-Ras, knockout of mutant but not WT K-Ras allele diminished the effects of PLX4032 in increasing ERK phosphorylation and DR5 expression (Fig. 3B). These results strongly suggest that PLX4032-induced ERK activation and DR5 upregulation is dependent on the presence of mutant Ras gene. In this study, we found that PLX4032 at 2.5 μM strongly increased ERK phosphorylation, but weakly elevated DR5 levels in HCT116 in contrast to its strong effects on both ERK activation and DR5 upregulation at 5 uM. Whether this suggests additional mechanism beyond ERK activation needs further investigation.

Moreover, we found that enforced expression of mutant K-Ras (12V) gene in 293T cells enhanced the basal levels of ERK phosphorylation and DR5 expression in comparison with those in cells carrying vector or WT K-Ras, both of which were further increased upon treatment with PLX4032 (Fig. 3C). In agreement, enforced expression of mutant K-Ras (12V) gene enhanced basal level DR5 promoter activity, which was also further increased by treatment with PLX4032 (Fig. 3D). These data further support the critical role of mutant Ras in mediating ERK activation and DR5 upregulation by PLX4032 in Ras-mutant cancer cells.

### PLX4032 induces CHOP/Elk1-mediated DR5 expression in Ras-mutant cancer cells

**Figure 4:**
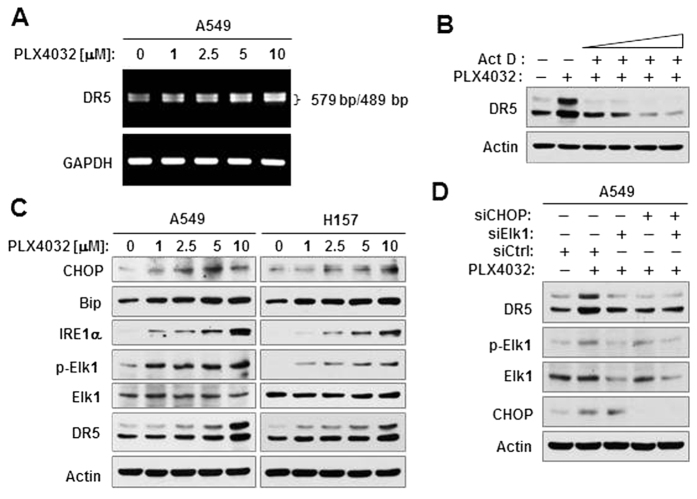
PLX4032 increases DR5 expression at the transcriptional level (**A,B**) through enhancement of CHOP/Elk1-mediated gene transcription (**C,D**). (**A)**, A549 cells were exposed to different concentrations of PLX4032 as indicated for 12 h and then harvested for preparation of total cellular RNA and subsequent RT-PCR to detect DR5 and GAPDH mRNAs. (**B)**, A549 cells were exposed to PLX4032 alone or in combination with different doses (1, 2.5, 5 and 7.5 μg/ml) of actinomycin D (Act D) for 12 h. (**C)**, the indicated cells were exposed to the given concentrations of PLX4032 for 12 h. (**D)**, A549 cells were transfected with the indicated siRNAs and then after 32 h exposed to 5 μM PLX4032 for an additional 12 h. After the aforementioned treatments (**B–D**), whole-cell protein lysates were prepared from the cells for detection of the indicated proteins with Western blotting.

It is known that regulation of DR5 expression can occur at transcriptional level^23^,^24^. Our previous studies have revealed that CHOP and Elk1 coordinately mediate DR5 gene transcription or expression induced by activation of Ras/Raf/MEK/ERK signaling^19^,^20^. Therefore we asked whether this is also the mechanism that accounts for DR5 upregulation by PLX4032 in Ras-mutant cells. We first showed that PLX4032 increased DR5 mRNA levels in a concentration-dependent manner in A549 cells (Fig. 4A). Moreover, the presence of the transcriptional inhibitor actinomycin D abolished DR5 induction by PLX4032 in the same cell line (Fig. 4B). These results indicate that PLX4032 induces DR5 expression at the transcriptional level. Following these experiments, we studied the effects of PLX4032 on Elk1 phosphorylation and expression of CHOP and other endoplasmic reticulum (ER) stress markers. As presented in Fig. 4C, PLX4032 increased the levels of CHOP, Bip, IRE1α and p-Elk1 in both A549 and H157 cells in a concentration-dependent manner, indicating that PLX4032 activates Elk1 and induces ER stress. We further examined whether CHOP and Elk1 are required for DR5 upregulation induced by PLX4032 by knocking down the expression of CHOP, Elk1 or both. We found that knockdown of CHOP, Elk1 or both abrogated the ability of PLX4032 to increase DR5 expression in A549 cells (Fig. 4D), indicating the dependency of DR5 induction by PLX4032 on both CHOP and Elk1.

### PLX4032 pre-treatment of Ras-mutant cancer cells confers sensitization to TRAIL-induced apoptosis

**Figure 5:**
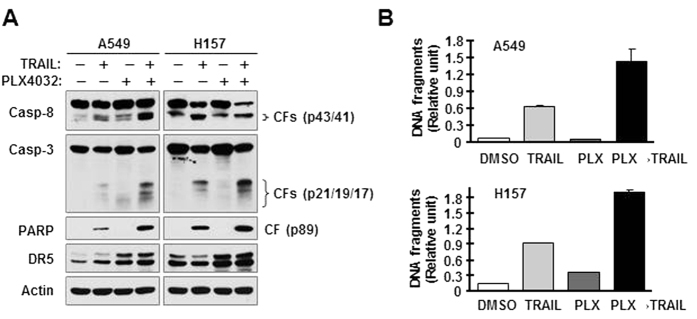
Pre-treatment of Ras-mutant cancer cells sensitizes them to TRAIL-induced apoptosis. The indicated cell lines were pre-exposed to 5 μM PLX4032 for 18 h. After removing medium containing the inhibitor, the cells were washed 3 times with medium and then fed with fresh medium containing 250 ng/ml (A549) or 150 ng/ml (H157) of TRAIL for additional 4 h. Protein cleavage was detected with Western blotting (**A**). DNA fragments were measured with the Cell Death Detection ELISA^Plus^ kit (**B**). The data represent means ± SDs of triplicate (**B**) determinations. CF, cleaved form.

Given that DR5 is a receptor for TRAIL, we speculated that B-Raf inhibition in Ras-mutant cancer cells will sensitize them to TRAIL-induced apoptosis due to a MEK/ERK-dependent increase in DR5 expression. Therefore, we pre-treated Ras-mutant cancer cells with PLX4032 overnight and then switched to TRAIL treatment. We detected much higher levels of cleaved caspase-8, caspase-3 and PARP in A549 and H157 cells pre-exposed to PLX4032, than in cells without PLX4032 pre-treatment (Fig. 5A). In agreement, we also detected much higher amounts of DNA fragments in A549 and H157 cells pre-treated with PLX4032, than in those without PLX4032 pre-treatment (Fig. 5B). These results together demonstrate that pre-exposure of Ras-mutant cancer cells to PLX4032 enhances TRAIL-induced apoptosis in these cells.

### Sensitization of Ras-mutant cancer cells to TRAIL-induced apoptosis by B-Raf inhibition is dependent on the activation of c-Raf/MEK/ERK signaling

**Figure 6:**
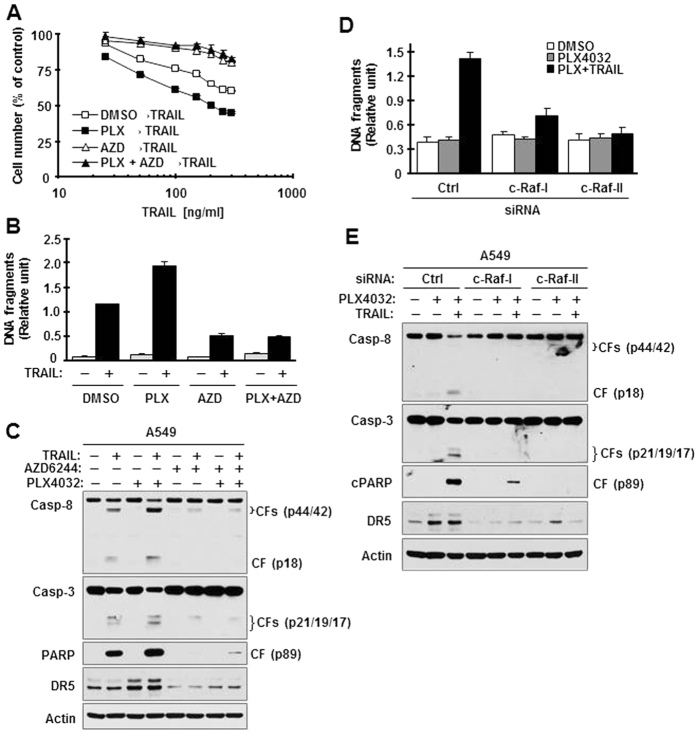
PLX4032-induced enhancement of TRAIL-induced apoptosis in Ras-mutant cancer cells can be abrogated by either MEK inhibition (**A–C**) or c-Raf inhibition (**D,E**). (**A–C**), A549 cells were pre-exposed to 5 μM of the tested agents alone or in combination for 12 h. After removing medium containing the inhibitors, the cells were washed 3 times with new medium and then fed with fresh medium containing the indicated concentrations of TRAIL for an additional 16 h (**A**) or 4 h (**B,C**). The cell numbers were estimated with the SRB assay (**A**). DNA fragments were measured with the Cell Death Detection ELISA^Plus^ kit (**B**). Protein cleavage was detected with Western blotting (**C***–***E)**, A549 cells were transfected with the given siRNAs for 32 h and then exposed to 5 μM of PLX4032 for 8 h and then switched to treatment with 250 ng/ml TRAIL. After an additional 4 h treatment, cells were harvested fro measurement of DNA fragments with the Cell Death Detection ELISA^Plus^ kit (**D**) and detection of protein cleavage with Western blotting (**E**). The data represent means ± SDs of four replicate (**A**) or triplicate (**B**,**D**) determinations. CF, cleaved form. PLX, PLX4032, AZD, AZD6244.

Finally, we determined whether B-Raf inhibition-induced sensitization of Ras-mutant cells to TRAIL-induced apoptosis is dependent on the activation of c-Raf/MEK/ERK signaling. Pre-exposure of A549 cells (approximately 18 h) to PLX4032 enhanced TRAIL-induced apoptosis, as evidenced by decreased cell survival (Fig. 6A), elevated DNA fragmentation (Fig. 6B), and increased cleavage of caspase-8, caspase-3 and PARP (Fig. 6C). However, these enhanced apoptosis-inducing effects were not observed in the cells pre-exposed to the combination of PLX4032 and AZD6244 (Fig. 6A–C). As expected, the presence of AZD6244 abolished DR5 upregulation induced by PLX4032 (Fig. 6C). Furthermore, we examined the importance of c-Raf in PLX4032-induced sensitization of cells to TRAIL-induced apoptosis. We detected increased DNA fragmentation and cleavage of caspase-8, caspase-3 and PARP in control siRNA-transfected A549 cells pre-treated with PLX4032 followed with TRAIL treatment, but not in c-Raf-siRNA-transfected cells experiencing the same treatment (Fig. 6D,E). In this study, we further validated c-Raf-dependent DR5 induction by PLX4032 since c-Raf knockdown blocked PLX4032-induced DR5 expression (Fig. 6E). Collectively, these results demonstrate that the sensitization of Ras-mutant cells to TRAIL-induced apoptosis by B-Raf inhibition is also dependent on the activation of c-Raf/MEK/ERK signaling.

## Discussion

This study demonstrates that B-Raf inhibition (e.g., with PLX4032 or dabrafenib) induces DR5 expression exclusively in Ras-mutant cancer cell lines. This effect is secondary to Ras/c-Raf-dependent activation of MEK/ERK signaling and eventual enhancement of CHOP and Elk1-mediated transcription of the DR5 gene. Hence the current results, together with our previously reported findings^19^,^20^,^21^, strongly indicate that Ras/Raf/MEK/ERK signaling-dependent and CHOP/Elk1-mediated gene transcription is a predominant mechanism for positive regulation of DR5 expression in cancer cells. In the current study, our results show that both Ras and c-Raf are required for PLX4032-induced ERK activation in Ras-mutant cancer cells, thus supporting the previous notion that B-Raf inhibition in Ras-mutant cancer cells induces c-Raf-dependent ERK activation^9^,^10^,^11^.

In the study, we noted that dabrafenib at high concentration ranges (e.g., > 1 μM) failed to activate ERK and increase DR5 expression (Fig. 1C). This compound has similar inhibitory activity against both B-Raf and c-Raf although it preferentially inhibits B-Raf (V600E)^25^. Therefore it is likely that dabrafenib, at high concentration ranges, inhibits c-Raf, resulting in blockage of c-Raf-dependent ERK activation and subsequent DR5 upregulation. Nonetheless, this finding again indicates a tight association between ERK activation and DR5 upregulaiton.

c-Raf-dependent rebound activation of MEK/ERK signaling in Ras-mutant cancer cells by B-Raf inhibitors is generally thought to be a mechanism that underlies resistance or accounts for an increased risk of secondary primary tumors^6^,^8^,^26^. Therefore the combination of B-Raf inhibition and MEK inhibition may be an effective way to block this rebound activation of MEK/ERK signaling^7^,^8^. Some clinical results in fact support this combinatorial approach^27^,^28^,^29^,^30^,^31^,^32^. However little attention has been paid to whether B-Raf inhibitor-induced rebound activation of MEK/ERK signaling in Ras-mutant cancer cells has any potential positive or beneficial impact on cancer treatment.

Clinical success with recombinant TRAIL or an agonistic DR5 antibody has not been demonstrated although the induction of apoptosis with these agents has emerged as an attractive cancer therapeutic strategy^17^,^33^,^34^. The current study has shown that pre-treatment of Ras-mutant cancer cell lines with PLX4032 sensitizes them to TRAIL-induced apoptosis; this effect is clearly due to c-Raf/MEK/ERK-dependent upregulation of DR5 expression because inhibition of MEK (e.g., with AZD6244) or c-Raf (e.g., with c-Raf siRNAs) abrogated the ability of PLX4032 not only to increase DR5 expression, but also to sensitize Ras-mutant cancer cells to TRAIL-induced apoptosis. This finding may suggest an innovative therapeutic strategy against Ras-mutant cancers through B-Raf inhibition with B-Raf selective inhibitors followed with a strategy that activates DR5-dependent or TRAIL/DR5-mediated apoptosis. This strategy may result in synthetic lethality of Ras-mutant cancer cells. Hence, further study in this direction is warranted.

Immunotherapy has become a very promising strategy in fight of cancers such as melanoma and lung cancer, and involves apoptotic death of cancer cells. Death ligand-induced apoptotic signaling, mainly by TRAIL from T cells, monocytes and dendritic cells, is one of the primary underlying mechanisms^12^,^15^,^35^. Therefore it is provocative to speculate that our findings may have a positive impact on the clinical efficacy of cancer immunotherapy. Whether B-Raf-targeted therapy followed with immunotherapy is a valid strategy against Ras-mutant cancers needs to be explored.

Induction of apoptosis by the interaction of endogenous TRAIL and DR5 is a critical immunosurveillance mechanism for our body to eliminate cancer cells^12^,^36^. Our findings of DR5 induction and sensitization of Ras-mutant cancer cells to TRAIL-induced apoptosis by B-Raf inhibition may have a positive impact in terms of enhancing immunosurveillance against Ras-mutant cancer cells. Moreover, we recently have shown that DR5 functions as an important suppressor of cancer cell invasion and metastasis^37^. Hence, whether B-Raf inhibition-induced MEK/ERK activation and DR5 expression has a long-term beneficial effect on Ras-mutant cancers deserves a further evaluation.

## Materials and Methods

### Reagents

PLX4032 and AZD6244 (selumetinib) were purchased from Selleckchem (Houston, TX). Dabrafenib was purchased from LC Laboratories (Woburn, MA). Human recombinant TRAIL was purchased from PeproTech, Inc. (Rocky Hill, NJ). c-Raf antibody was purchased from Cell Signaling (Danvers, MA). Other antibodies, WT and mutant K-Ras (12V) were described previously^19^,^20^.

### Cell lines

The B-Raf or Ras mutation status of the tested human cancer cell lines is summarized in Table 1. Except for A549, HCT116, H1299 and BCPAP, other cell lines were not authenticated. HCT116 (K-Ras; wt/mut), HCT116 (K-Ras; del/mut) and HCT116 (K-Ras; wt/del) were generously provided by Dr. B. Vogelstein (Johns Hopkins University School of Medicine, Baltimore, MD). They were grown in RPMI 1640 medium supplemented with 5% fetal bovine serum at 37 °C in a humidified atmosphere consisting of 5% CO_2_.

### Cell survival assay

Cells were seeded in 96-well cell culture plates and on the second day exposed to different agents. Viable cell numbers were then estimated with sulforhodamine B (SRB) assay^38^.

### Detection of apoptosis

A Cell Death Detection ELISA^Plus^ kit (Roche Molecular Biochemicals, Indianapolis, IN) was used to measure a poptosis according to the manufacturer’s instructions. Western blot analysis was used to detect cleavage of caspase and PARP for additional indications of apoptosis.

### Western blot analysis

Preparation of whole-cell protein lysates and Western blotting were the same as described previously^19^.

### Detection of cell surface DR5

Detection of cell surface DR5 expression with flow cytometry was described previously^39^.

### Detection of DR5 mRNA expression

RT-PCR as described previously^39^,^40^ was used for detection of DR5 and GAPDH internal control mRNAs.

### Detection of DR5 promoter activity

The reporter construct containing a 552-bp 5-flanking region of the DR5 gene, pGL3-DR5 (−552)-luc was generously provided by Dr. H-G Wang (Penn State University, PA)^23^ and used in our previous studies^19^,^20^,^39^. Plasmid transfection and luciferase assay were conducted as described previously^39^.

### Gene knockdown using siRNA

Gene knockdown was achieved by transfecting siRNA with HiPerFect transfection reagent (Qiagen, Valencia, CA) following the manufacturer’s instruction. Control (siCtrl; i.e., non-silencing), K-Ras, CHOP and Elk1 siRNAs were described in our previous study^20^. ERK1/2 (#6560) and c-Raf siRNAs (#12256 and #12342) were purchased from Cell Signaling. Gene knockdown efficiencies were evaluated by Western blot analysis.

## Additional Information

**How to cite this article**: Oh, Y.-T. *et al*. Paradoxical activation of MEK/ERK signaling induced by B-Raf inhibition enhances DR5 expression and DR5 activation-induced apoptosis in Ras-mutant cancer cells. *Sci. Rep.* **6**, 26803; doi: 10.1038/srep26803 (2016).

## Figures and Tables

**Table 1:** Ras and B-Raf mutation status of the cancer cell lines used in this study.

**Cell line**	**Ras mutation**	**B-Raf mutation**	**Cancer type**
A549	K-Ras (G12S)	WT	Lung
H1299	N-Ras (Q61K)	WT	Lung
H157	K-Ras (G12R)	WT	Lung
Calu-1	K-Ras (G12C)	WT	Lung
HCT116	K-Ras (G13D)	WT	Colon
HCT15	K-Ras (G13D)	WT	Colon
Cal-62	K-Ras (G12R)	WT	Thyroid
A375	WT	V600E	Melanoma
SK-MEL-5	WT	V600E	Melanoma
UACC-257	WT	V600E	Melanoma
M14	WT	V600E	Melanoma
BCPAP	WT	V600E	Thyroid
HT29	WT	V600E	Colon
H226	WT	WT	Lung
H596	WT	WT	Lung
H1975	WT	WT	Lung
H1650	WT	WT	Lung
PC-9	WT	WT	Lung

The information listed in this table was obtained from Sanger (http://cancer.sanger.ac.uk/cell_lines) or provided by Dr. J. D. Minna (University of Texas Southwestern Medical Center, Dallas, TX).

## References

[b1] Holderfield M., Deuker M. M., McCormick F. & McMahon M. Targeting RAF kinases for cancer therapy: BRAF-mutated melanoma and beyond. Nat Rev Cancer 14, 455–467 (2014). 24957944 10.1038/nrc3760 PMC4250230

[b2] Huang T., Karsy M., Zhuge J., Zhong M. & Liu D. B-Raf and the inhibitors: from bench to bedside. J Hematol Oncol 6, 30 (2013). 23617957 10.1186/1756-8722-6-30 PMC3646677

[b3] Li H. F. . Recent advances in the research and development of B-Raf inhibitors. Curr Med Chem 17, 1618–1634 (2010). 20345352 10.2174/092986710791111242

[b4] Rheault T. R. . Discovery of Dabrafenib: A Selective Inhibitor of Raf Kinases with Antitumor Activity against B-Raf-Driven Tumors. ACS medicinal chemistry letters 4, 358–362 (2013). 24900673 10.1021/ml4000063 PMC4027516

[b5] Akinleye A., Furqan M., Mukhi N., Ravella P. & Liu D. MEK and the inhibitors: from bench to bedside. J Hematol Oncol 6, 27 (2013). 23587417 10.1186/1756-8722-6-27 PMC3626705

[b6] Haarberg H. E. & Smalley K. S. Resistance to Raf inhibition in cancer. Drug discovery today. Technologies 11, 27–32 (2014). 24847650 10.1016/j.ddtec.2013.12.004 PMC4031441

[b7] Lito P., Rosen N. & Solit D. B. Tumor adaptation and resistance to RAF inhibitors. Nat Med 19, 1401–1409 (2013). 24202393 10.1038/nm.3392

[b8] Holderfield M., Nagel T. E. & Stuart D. D. Mechanism and consequences of RAF kinase activation by small-molecule inhibitors. Br J Cancer 111, 640–645 (2014). 24642617 10.1038/bjc.2014.139 PMC4134487

[b9] Hatzivassiliou G. . RAF inhibitors prime wild-type RAF to activate the MAPK pathway and enhance growth. Nature 464, 431–435 (2010). 20130576 10.1038/nature08833

[b10] Poulikakos P. I., Zhang C., Bollag G., Shokat K. M. & Rosen N. RAF inhibitors transactivate RAF dimers and ERK signalling in cells with wild-type BRAF. Nature 464, 427–430 (2010). 20179705 10.1038/nature08902 PMC3178447

[b11] Heidorn S. J. . Kinase-dead BRAF and oncogenic RAS cooperate to drive tumor progression through CRAF. Cell 140, 209–221 (2010). 20141835 10.1016/j.cell.2009.12.040 PMC2872605

[b12] Falschlehner C., Schaefer U. & Walczak H. Following TRAIL’s path in the immune system. Immunology 127, 145–154 (2009). 19476510 10.1111/j.1365-2567.2009.03058.x PMC2691779

[b13] Gonzalvez F. & Ashkenazi A. New insights into apoptosis signaling by Apo2L/TRAIL. Oncogene 29, 4752–4765 (2010). 20531300 10.1038/onc.2010.221

[b14] Lim B. . Targeting TRAIL in the treatment of cancer: new developments. Expert opinion on therapeutic targets, 19, 1171–1185 (2015). 26004811 10.1517/14728222.2015.1049838

[b15] Smyth M. J. . Nature’s TRAIL–on a path to cancer immunotherapy. Immunity 18, 1–6 (2003). 12530970 10.1016/s1074-7613(02)00502-2

[b16] Yang A., Wilson N. S. & Ashkenazi A. Proapoptotic DR4 and DR5 signaling in cancer cells: toward clinical translation. Curr Opin Cell Biol 22, 837–844 (2010). 20813513 10.1016/j.ceb.2010.08.001

[b17] Bellail A. C., Qi L., Mulligan P., Chhabra V. & Hao C. TRAIL agonists on clinical trials for cancer therapy: the promises and the challenges. Rev Recent Clin Trials 4, 34–41 (2009). 19149761 10.2174/157488709787047530

[b18] Falschlehner C., Ganten T. M., Koschny R., Schaefer U. & Walczak H. TRAIL and other TRAIL receptor agonists as novel cancer therapeutics. Adv Exp Med Biol 647, 195–206 (2009). 19760076 10.1007/978-0-387-89520-8_14

[b19] Oh Y. T. . ERK/ribosomal S6 kinase (RSK) signaling positively regulates death receptor 5 expression through co-activation of CHOP and Elk1. J Biol Chem 285, 41310–41319 (2010). 21044953 10.1074/jbc.M110.153775 PMC3009856

[b20] Oh Y. T. . Oncogenic Ras and B-Raf proteins positively regulate death receptor 5 expression through co-activation of ERK and JNK signaling. J Biol Chem 287, 257–267 (2012). 22065586 10.1074/jbc.M111.304006 PMC3249076

[b21] Oh Y. T. . Inhibition of B-Raf/MEK/ERK signaling suppresses DR5 expression and impairs response of cancer cells to DR5-mediated apoptosis and T cell-induced killing. Oncogene 35, 459–467 (2015). 25867065 10.1038/onc.2015.97 PMC4604000

[b22] Downward J. & Targeting R. A. F.: trials and tribulations. Nat Med 17, 286–288 (2011). 21383738 10.1038/nm0311-286

[b23] Yamaguchi H. & Wang H. G. CHOP is involved in endoplasmic reticulum stress-induced apoptosis by enhancing DR5 expression in human carcinoma cells. J Biol Chem 279, 45495–45502 (2004). 15322075 10.1074/jbc.M406933200

[b24] Wu G. S. . KILLER/DR5 is a DNA damage-inducible p53-regulated death receptor gene. Nature genetics 17, 141–143 (1997). 9326928 10.1038/ng1097-141

[b25] King A. J. . Dabrafenib; preclinical characterization, increased efficacy when combined with trametinib, while BRAF/MEK tool combination reduced skin lesions. Plos One 8, e67583 (2013). 23844038 10.1371/journal.pone.0067583 PMC3701070

[b26] Gibney G. T., Messina J. L., Fedorenko I. V., Sondak V. K. & Smalley K. S. Paradoxical oncogenesis–the long-term effects of BRAF inhibition in melanoma. Nat Rev Clin Oncol 10, 390–399 (2013). 23712190 10.1038/nrclinonc.2013.83 PMC3983565

[b27] Flaherty K. T. . Combined BRAF and MEK inhibition in melanoma with BRAF V600 mutations. N Engl J Med 367, 1694–1703 (2012). 23020132 10.1056/NEJMoa1210093 PMC3549295

[b28] Grob J. J. . Comparison of dabrafenib and trametinib combination therapy with vemurafenib monotherapy on health-related quality of life in patients with unresectable or metastatic cutaneous BRAF Val600-mutation-positive melanoma (COMBI-v): results of a phase 3, open-label, randomised trial. Lancet Oncol 16, 1389–1398 (2015). 26433819 10.1016/S1470-2045(15)00087-X

[b29] Long G. V. . Dabrafenib and trametinib versus dabrafenib and placebo for Val600 BRAF-mutant melanoma: a multicentre, double-blind, phase 3 randomised controlled trial. Lancet 386, 444–451 (2015). 26037941 10.1016/S0140-6736(15)60898-4

[b30] Robert C. . Improved overall survival in melanoma with combined dabrafenib and trametinib. N Engl J Med 372, 30–39 (2015). 25399551 10.1056/NEJMoa1412690

[b31] Larkin J. . Combined vemurafenib and cobimetinib in BRAF-mutated melanoma. N Engl J Med 371, 1867–1876 (2014). 25265494 10.1056/NEJMoa1408868

[b32] Johnson D. B. . Combined BRAF (Dabrafenib) and MEK inhibition (Trametinib) in patients with BRAFV600-mutant melanoma experiencing progression with single-agent BRAF inhibitor. J Clin Oncol 32, 3697–3704 (2014). 25287827 10.1200/JCO.2014.57.3535 PMC4226803

[b33] Mocellin S. Targeting death receptors to fight cancer: from biological rational to clinical implementation. Curr Med Chem 17, 2713–2728 (2010). 20586721 10.2174/092986710791859342

[b34] Lemke J., von Karstedt S., Zinngrebe J. & Walczak H. Getting TRAIL back on track for cancer therapy. Cell Death Differ 21, 1350–1364 (2014). 24948009 10.1038/cdd.2014.81 PMC4131183

[b35] Hersey P. & Zhang X. D. Treatment combinations targeting apoptosis to improve immunotherapy of melanoma. Cancer Immunol Immunother 58, 1749–1759 (2009). 19551381 10.1007/s00262-009-0732-5 PMC11030855

[b36] Johnstone R. W., Frew A. J. & Smyth M. J. The TRAIL apoptotic pathway in cancer onset, progression and therapy. Nat Rev Cancer 8, 782–798 (2008). 18813321 10.1038/nrc2465

[b37] Oh Y. T. . Suppression of death receptor 5 enhances cancer cell invasion and metastasis through activation of caspase-8/TRAF2-mediated signaling. Oncotarget 6, 41324–41338 (2015). 26510914 10.18632/oncotarget.5847 PMC4747408

[b38] Sun S. Y. . Differential effects of synthetic nuclear retinoid receptor-selective retinoids on the growth of human non-small cell lung carcinoma cells. Cancer Res 57, 4931–4939 (1997). 9354460

[b39] Sun S. Y. . The Farnesyltransferase Inhibitor Lonafarnib Induces CCAAT/Enhancer-binding Protein Homologous Protein-dependent Expression of Death Receptor 5, Leading to Induction of Apoptosis in Human Cancer Cells. J Biol Chem 282, 18800–18809 (2007). 17493934 10.1074/jbc.M611438200

[b40] Dhandapani L., Yue P., Ramalingam S. S., Khuri F. R. & Sun S. Y. Retinoic acid enhances TRAIL-induced apoptosis in cancer cells by upregulating TRAIL receptor 1 expression. Cancer Res 71, 5245–5254 (2011). 21685476 10.1158/0008-5472.CAN-10-4180 PMC3151668

